# Effect of the prism-interprisms three-dimension spatial microstructure on the enamel bond strength

**DOI:** 10.1186/s12903-023-03599-3

**Published:** 2023-11-13

**Authors:** Chaoyang Wang, Jianhao Xu, Jingqiu Xu, Songwen Deng, Baiping Fu, Ling Zhang

**Affiliations:** 1 Stomatology Hospital, School of Stomatology, Zhejiang University School of Medicine, Zhejiang Provincial Clinical Research Center for Oral Diseases, Key Laboratory of Oral Biomedical Research of Zhejiang Province, Cancer Center of Zhejiang University, Engineering Research Center of Oral Biomaterials and Devices of Zhejiang Province, Hangzhou, 310006 China; 2 School of Stomatology, Zhejiang University School of Medicine, Hangzhou, China

**Keywords:** Prism-interprisms, Microstructure, Enamel bonding, Dentistry, Bond strength

## Abstract

The prism-interprisms level of the enamel hierarchical microstructure is the largest degree of structural variation and most sophisticated structural adaptation. We studied the effect of the prism-interprisms three-dimension spatial microstructure on the enamel bond strength. We prepared 11 groups of enamel segments: longitudinally sectioned segments with or without a 45-degree bevel (group = 2), horizontally sectioned segments with or without a 45-degree bevel of three regions (the incisal, middle, and cervical) (group = 6), and tangential (labial) sectioned segments of three regions (the incisal, middle, and cervical) (group = 3). The finished surface of each segment was observed by scanning electric microscopy (SEM) before treatment with four self-etch adhesive systems and applied with four corresponding composite resins. Resin-bonded enamel samples were prepared in beams for microtensile bond strength (MTBS) tests. The results were analyzed with a three-way ANOVA followed by Tukey’s post-hoc HSD multiple comparisons procedure. SEM observations revealed complex arrangements of prisms and interprisms. MTBS measurement showed that the longitudinally sectioned group had the lowest value, without significant differences between the groups with or without 45-degree bevel. Combining SEM observations and MTBS measurements, the prism-interprisms microstructure varied with the incisor regions, and different prism-interprisms microstructures allowed diverse sectioned surfaces, which could affect the enamel bonding.

## Introduction

The enamel bonding of cavity margins is closely related to the success and durability of composite restorations [1]. In a dental practice, cavities prepared after dental caries removal have several walls and floors. The margin enamel of cavity walls was cut in an incisal-cervical (longitudinal) direction or labial-lingual or distal-mesial (horizontal) direction [2, 3]. Tooth enamel is an anisotropic material with its prismatic, rod-like apatite morphology [4–11]. Because of this structural anisotropy, variation in enamel bonding sites affects the enamel bond strength [12–16].

Limited studies focus on the effect of the prism-interprisms three-dimension spatial microstructure on the enamel bond strength [13–15, 17, 18]. Ikeda et al. [17] and Carvalho et al. [18] revealed lower micro tensile bond strengths (MTBS) in specimens stressed perpendicular to the prism long axis than in specimens stressed parallel to the prism axis. However, only one or two section directions have been tested, ignoring the regional variation of the enamel microstructure. Shimada et al. [12–15] reported that the bonded surfaces perpendicular to the prisms showed high bonding strength [12–15]. But only one or two tooth regions and two adhesives were studied. However, the enamel is characterized by a complex three-dimensional microstructure. Enamel microstructure like Hunter-Schreger Bands (HSBs) consisted of decussating prisms groups could cause variable micrographs of bonded surfaces in different tooth regions [19–24]. The enamel bonding might be influenced by the HSB. But insufficient evidence has corroborated how the HSBs improve enamel bonding.

Enamel bevels are usually recommended for peripheral enamel margins of resin restorations [25, 26]. Bevel preparation of enamel margins can remove weakly supported or unsupported enamel margins and improve esthetics via the color transition at enamel-resin interfaces [25]. Bevel preparation also increases the surface area for enamel bonding. However, beveling of enamel does not result in greatly increased enamel bond strength or improved margin quality [25, 26], and it is unclear whether enamel margins should be beveled or not.

Above all, we hypothesized that different regions and section directions of bovine incisors will not influence the enamel MTBS.

## Materials and methods

### Sample preparations

**Fig. 1:**
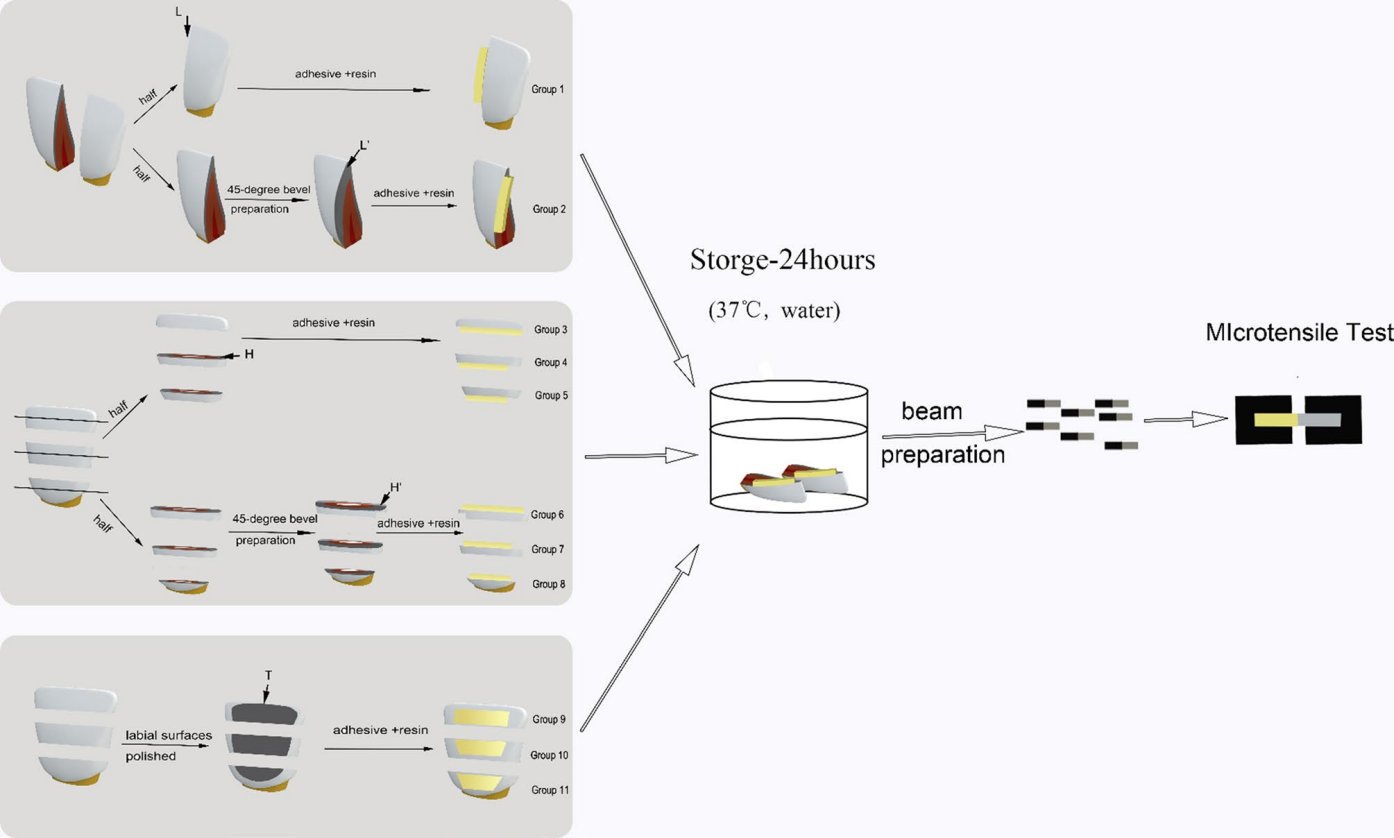
Schematic illustrations of the bonded sample preparations. Longitudinal segments: The crowns of bovine incisors are cut in a longitudinal (labio-lingual) direction through the middle of the crown, and then half of the segments were prepared with a 45-degree mesial bevel to obtain another group of longitudinal segments with a 45-degree bevel. Horizontal segments: the crowns of bovine incisors are horizontally sectioned (solid lines) along the incisal and cervical thirds for horizontal segments and further horizontally cut in two (dotted lines), and then half of the horizontal segments were prepared with a 45-degree cervical bevel to obtain another group of horizontal segments with a 45-degree bevel. Tangential segments: the crowns of bovine incisors are sectioned along the incisal and cervical thirds to obtain tangential (labial) segments, and then the labial surface of each tangential segment was polished for later observation and enamel bonding. Four self-etch adhesives are applied to the enamel surfaces and composite resin is placed. After water storage at 37°C for 24 h, all samples were prepared for Mcro tensile test. (L, H and T: longitudinal, horizontal and tangential section-directions of the bovine incisors, respectively; L’ and H’: L and H sections with a 45-degree bevel, respectively; D: dentin, E: enamel, R: composite resin)

In order to obtained enough enamel adhesive surfaces, twenty caries-free bovine mandibular incisors stored in 1 wt% of thymol solution at 4 °C were used within two months after extraction. This research protocol was per-formed in accordance with the international Ethical Guideline and Declaration of Helsinki and approved by the ethics committee of Zhejiang University School of Stomatology (World Health Organization, 2002; World Medical Association, 2008). The bovine mandibular incisors were sectioned using a slow-speed diamond saw (Isomet1000 Precision Cutter; Buehler, Lake Bluff, IL, USA) under continuous water cooling. Eleven groups were prepared as follows (Fig. 1): (a) eight incisors were bisected longitudinally through the middle of the crown. The middle region of the labial surface was used to obtain longitudinal segments (Group 1). Half of the longitudinal segments were further prepared with a 45-degree bevel along the whole enamel thickness (Group 2). (b) Another eight incisors were trisected horizontally to produce three horizontal segments, and then each horizontal segment was further horizontally sectioned into two equal pieces. Half of horizontal pieces of incial/middle/cervical third were remained as Group 3~5. The rest half of three regions were further prepared with a 45-degree bevel along the whole enamel thickness (Group 6 ~ 8). (c) The last four incisors were trisected horizontally to obtain incisal, middle, and cervical segments. Each segment was further polished on the labial (tangential) surface (Group 9 ~ 11).

The sectioned enamel and labial surfaces were polished serially with 300-, 800-, and 1200-grit silicon-carbide papers by the grinding and polishing machine (260E Polishing machine; WeiYi, Laizhou, China) under running water. In order to reduce the chemical bonding [27, 28], we used four self-etch adhesive systems without the most effective Functional monomer-10-methacryloyloxydecyl dihydrogenphosphate (MDP). Four self-etch adhesive systems (Xeno III, Dentsply, Konstanz, Germany; iBond, Heraeus Kulzer, Hanau, Germany; G Bond, GC, Tokyo, Japan and Adper Easy One, 3 M ESPE, St Paul, USA) and four corresponding composite resins (Spectrum TPH 3,Dentsply, Konstanz, Germany; Venus, Heraeus Kulzer, Hanau, Germany; Gradia Direct, GC, Tokyo, Japan and Filtek Z350, 3 M ESPE, St Paul, USA) were applied to the polished enamel surfaces strictly according manufacturer’s instructions (Fig. 1). Light curing was performed with a power output of 1500 mW/cm^2^ (Radii plus SDI; Victoria, Australia). The adhesive ingredients, composite resins, and application steps are summarized in Table 1. After water storage at 37 °C for 24 h, all samples were perpendicularly sectioned through the resin-enamel interfaces using water cooling to obtain 1 mm slices. The slices were prepared into multiple regular beams (1 mm × 1 mm × 8 mm) for MTBS tests. Each subgroup comprised 10–17 beams.

**Table 1:** Adhesive systems and composite resins used in this study

Products (manufacturers, batch#)		Ingredients	Application Procedures	Codes
Adper Easy One Filtek Z350 (3 M ESPE, Germany)	457,979	HEMA, Bis-GMA, water, phosphoric acid-methacryloxy-hexylesters, ethanol, silane-treated silica, HDDMA, copolymer of acrylic and itaconic acid, DMAEMA, phosphine oxide, CQ	Apply the adhesive for 20 s, gently air-blow for 5 s, light-cure for 10 s	AEO
	N304250	Bis-GMA, UDMA, TEGDMA, Bis-EMA resins	Place two 2-mm increments, light-cure each for 40 s	
GC BOND GRADIA DIRECT (GC Co, Japan)	1,109,231	4-MET, phosphate acid, UDMA, silica, photoinitiator, water, acetone	Apply the adhesive for 10 s, strongly air-blow for 5 s, light-cure for 10 s	GC
	1,201,051	UDMA, silica powder, Alumino-silicate glass, organic filler	Place two 2-mm increments, light-cure each for 20 s	
i Bond Venus (Heraeus, Germany)	010113	4-MET, UDMA, TEGDMA, acetone, glutaraldehyde, photo initiator, stabilizer, purified water	Apply and leave undisturbed for 30 s, thoroughly air dry, light-cure for 20 s.	IB
	311	Bis-GMA, TEGDMA, organic filler, Bis-EMA resins	Place two 2-mm increments, light-cure each for 20 s	
Xeno-III Spectrum TPH3 (Dentsply, Germany)	LiquidA:1,105,000,297 LiquidB:1,105,000,298	HEMA, ethanol, water, aerosil, BHT Pyro-EMA, PEM-F, UDMA, CQ, BHT, ethyl-4-dimethylaminobenzoate (co-intiator)	Apply a mixture of liquid A and B (1:1) for 20 s, gently air-dry, light-cure for 20 s.	Xeno-III
	1,109,000,719	~ 1 μm conventional glass fillers a methacrylate modified polysiloxane, a dimethacrylate resin	Place two 2-mm increments, light-cure each for 20 s	

Abbreviations: HEMA:2-hydroxyethyl methacrylate; Bis-GMA: Bisphenol-A diglycidyl methacrylate; HDDMA: 1,6-Hexanediol dimethacrylate DMAEMA: dimethylaminoethyl methacrylate; CQ: camphorquinone. UDMA: urethane dimethacrylate or 1,6-di (methacryloyloxyethylcarbamoyl)-3,30,5-trimethylhexane; TEGDMA: triethylene glycol dimethacrylate; Bis-EMA: Bisphenol A Ethoxylate Dimethacrylate; 4-MET: 4-methacryloylox ethyl trimellitic acid; BHT: butylhydroxytoluene or butylated hydroxytoluene or 2,6-di-(tert-butyl)-4-methylphenol (inhibitor);Pyro-EMA: tetramethacryloyloxyethyl pyrophosphate; PEM-F: pentamethacryloyloxyethylcyclohexaphosphazene monofluoride

### MTBS measurements

The enamel MTBS tests were performed in a Micro Tensile Tester (Bisco; Schaumburg, IL, USA) at a crosshead speed of 1 mm/min until failure [29]. After the failure, the exact dimension of fractured surface was measured with a vernier caliper (Hangzhou Qiantangjiang Measuring Tools, Hangzhou, China) with an accuracy of 0.01 mm. MTBS was calculated in MegaPascals (MPa). The specimens of the pre-testing failures were only recorded in numbers, but not calculated in the total data.

### Failure analysis

The failure modes were determined with stereomicroscopy (Leica MZ APO; Leica Microsystems, Heerbrugg, Switzerland) at 50-fold magnification. There were four failure modes: cohesive failure in enamel or resin, adhesive failure, and mixed failure [30, 31].

### Statistics

Statistical analysis was conducted using IBM SPSS 26.0(IBM; New York, USA). The data were tested with the Kolmogorow-Smirnov for normal distribution. A three-way ANOVA followed by LSD post hoc HSD multiple comparisons were performed to analyze the enamel MTBS among different section directions, regions and adhesives. Failure mode data were analyzed using Chi square tests. The statistical significance level was set at 0.05.

### Scanning electron microscopy (SEM)

One sample from each group was observed before adhesive was applied and placed with composite on SEM (Ultra55; Zeiss, Oberkochen, Germany). Other enamel samples were immersed in 1 mol/L hydrochloric acid solution for 10 s, dehydrated in ascending ethanol concentrations and platinum-sputtered before SEM observations. SEM analysis was performed under 4.5–6 KV at a working distance of 5–8.5 mm in the secondary electron mode. The fracture surfaces of representative surfaces of each subgroup were prepared and analyzed.

## Results

### MTBS

There was a statistically significant interaction between section directions, regions, and adhesive used (*P* < 0.05). The mean MTBS values of all groups are summarized in Table 2. The adhesive types did not significantly improve the enamel MTBS (*P* > 0.05). The enamel MTBS values of the incisal region were significantly lower than those of the middle and cervical regions (*P <* 0.05). The longitudinally sectioned resin-bonded enamel samples showed the lowest enamel MTBS among all groups. The 45-degree bevel had no significant effect on these samples (*P* > 0.05). The horizontally sectioned resin-bond samples showed the highest enamel MTBS (*P <* 0.05). However, MTBS of horizontally sectioned resin-bonded samples with a 45-degree bevel was lower. The tangentially sectioned resin-bonded samples had enamel MTBS similar to that of horizontally sectioned resin-bonded samples with a 45-degree bevel (*P >* 0.05).

**Table 2:** Enamel MTBS in MPa (Mean ± SD, (n + f)) analyzed by three-way ANOVA followed by Tukey’s post hoc HSD multiple comparisons

Group	Section Directions	Regions	Self-etch Adhesives			
			AEO	GC	IB	Xeno III
	Longitudinal (no bevel)	Incisal third				
1		Middle third	6.99 ± 2.09(11)^A,a^	4.05 ± 0.91(11 + 5)^A,a^	6.12 ± 3.38 (10 + 7)^A,ab^	5.89 ± 2.13(10 + 7)^A,ab^
		Cervical third				
	Longitudinal (45º- bevel)	Incisal third				
2		Middle third	7.54 ± 1.06(11)^A,a^	10.06 ± 3.02(13 + 3)^A,a^	7.38 ± 2.14(10)^A,a^	8.55 ± 1.61(10)^A,a^
		Cervical third				
3	Horizontal (no bevel)	Incisal third	16.12 ± 5.41(11)^B,b^	17.50 ± 5.35(10)^B,b^	15.44 ± 5.43(10)^B,b^	18.11 ± 2.93(10 + 1)^B,b^
4		Middle third	18.31 ± 4.01(10 + 3)^B,c^	17.13 ± 4.43(10)^B,c^	19.48 ± 6.79(11)^B,c^	20.57 ± 9.21(12)^B,c^
5		Cervical third	21.97 ± 3.70(10 + 4)^B,c^	17.65 ± 3.95(10)^B,c^	15.41 ± 4.91(10)^B,c^	21.73 ± 8.52(11)^B,c^
6	Horizontal (45º- bevel)	Incisal third	14.51 ± 3.19(11)^C,b^	12.59 ± 5.10(10)^C,b^	16.25 ± 3.79(10)^C,b^	16.50 ± 6.49(10 + 1)^C,b^
7		Middle third	14.90 ± 6.25(10)^C,c^	16.31 ± 4.58(10)^C,c^	20.54 ± 5.43(10)^C,c^	13.42 ± 4.73(10)^C,c^
8		Cervical third	18.55 ± 4.07(10 + 4)^C,c^	15.16 ± 4.56(10)^C,c^	21.31 ± 7.48(10)^C,c^	17.55 ± 5.82(10)^C,c^
9	Tangential (Labial)	Incisal third	9.12 ± 3.72(12)^C,b^	15.92 ± 3.31(10)^C,b^	20.86 ± 4.07(11)^C,b^	15.64 ± 3.06(11)^C,b^
10		Middle third	12.70 ± 2.52(11)^C,c^	14.89 ± 3.72(14)^C,c^	20.97 ± 5.46(10)^C,c^	17.75 ± 5.06(11)^C,c^
11		Cervical third	11.09 ± 3.63(10)^C,c^	12.77 ± 3.62(12)^C,c^	22.90 ± 7.03(12)^C,c^	21.95 ± 3.85(11)^C,c^

n + f: indicates the number of specimens and the number of pre-testing specimens

Different superscript uppercase letters denote statistically significant different between different sections

Different superscript lowercase letters denote statistically significant different between different regions

### Failure analysis

**Fig. 2:**
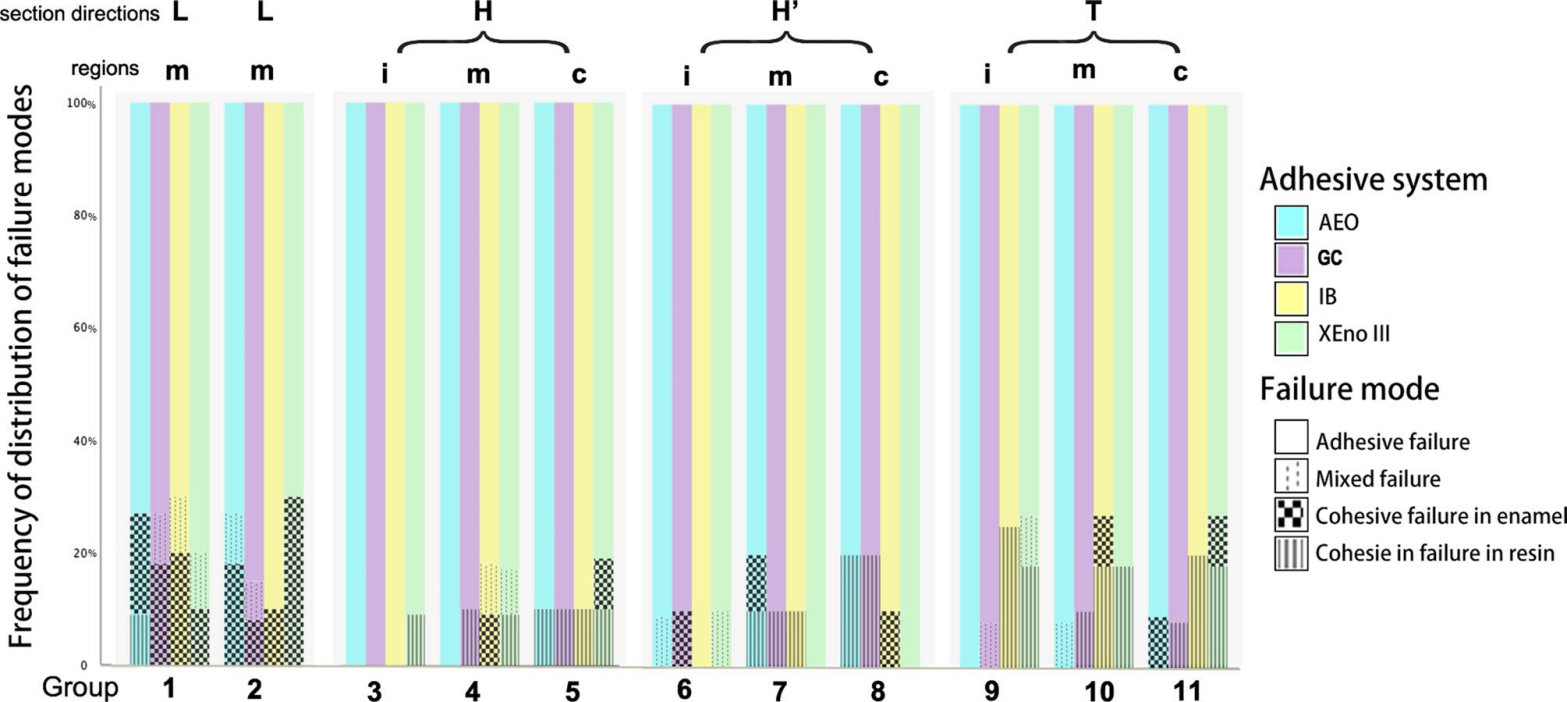
Frequency distributions of the failure modes according to cutting directions, beveling and adhesive system. (L, H and T: indicate the longitudinal, horizontal and tangential cutting directions of the bovine incisors, respectively; L’ and H’: indicate L and H sections with a 45-degree bevel, respectively. i, m and c: indicate the incisal, middle cervical thirds, respectively)

The predominant failure mode of all groups was adhesive failure (*P* < 0.001) (Fig. 2; Table 3). Most cohesive failures in enamel occurred in the longitudinally sectioned resin-bonded enamel samples with or without a 45-degree bevel. Cohesive failures in resin occurred in most groups at a range of 0 ~ 25%. Mixed failures occurred sporadically.

**Table 3:** The fracture mode of each subgroup

Group	Section Directions	Regions	Self-etch Adhesives															
			AEO				GC				IB				Xeno III			
		Facture mode	A	M	E	R	A	M	E	R	A	M	E	R	A	M	E	R
	Longitudinal (no bevel)	Incisal third																
1		Middle third	8	0	2	1	8	1	2	0	7	1	2	0	8	1	1	0
		Cervical third																
	Longitudinal (45º- bevel)	Incisal third																
2		Middle third	8	1	2	0	11	1	1	0	9	0	1	0	7	0	3	0
		Cervical third																
3	Horizontal (no bevel)	Incisal third	11	0	0	0	10	0	0	0	10	0	0	0	9	0	0	1
4		Middle third	10	0	0	0	9	0	0	1	9	1	1	0	10	1	0	1
5		Cervical third	9	0	0	1	9	0	0	1	9	0	0	1	9	0	1	1
6	Horizontal (45º- bevel)	Incisal third	10	1	0	0	9	0	1	0	10	0	0	0	9	1	0	0
7		Middle third	8	0	1	1	9	0	0	1	9	0	0	1	10	0	0	0
8		Cervical third	8	0	0	2	8	0	0	2	9	0	1	0	10	0	0	0
9	Tangential (Labial)	Incisal third	11	1	0	0	9	0	0	1	8	0	1	2	9	0	0	2
10		Middle third	10	0	1	0	12	0	0	1	8	0	0	2	8	0	1	2
11		Cervical third	10	0	0	0	11	1	0	0	9	0	0	3	8	1	0	2

A: Adhesive failure; M: Mixed failure; E: Cohesive failure in enamel; R: Cohesive failure in resin

### Sectioned enamel surfaces

SEM micrographs revealed that the enamel microstructure varied within different regions and section directions of the tooth.

**Fig. 3:**
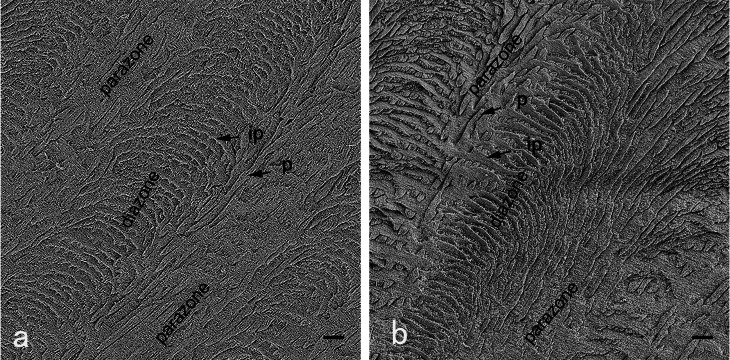
SEM micrographs of the longitudinal enamel sections with or with a 45-degree bevel in middle regions. The prisms (p) are shown as HSBs with two decussating prism groups. The longitudinally cut prisms (parazones) and transversely cut prisms (diazones) alternate at intervals. Abundant interprisms (ip) are alternated with the prisms layer by layer. (bar = 10 μm, 1000×)

**Fig. 4:**
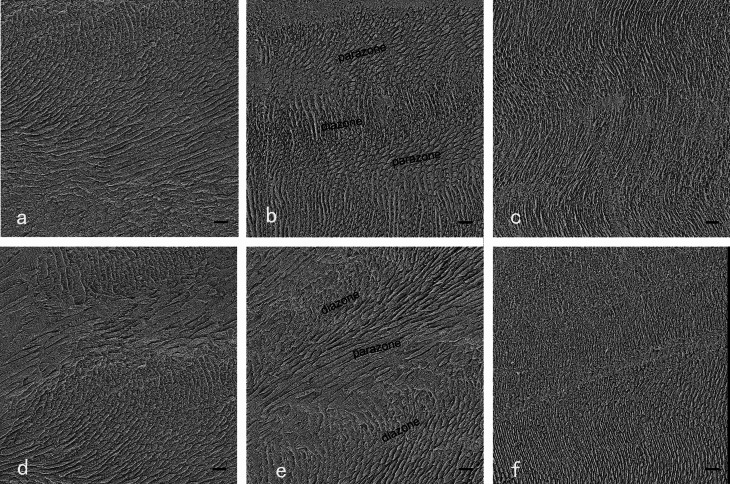
SEM micrographs of horizontal enamel sections in three regions. a,b,c shows SEM micrographs of horizontal enamel sections. d,e,f shows SEM micrographs of horizontal enamel sections with a 45-degree bevel. a: Incisal region: prisms are almost transversely cut. b: Middle region: The prisms appear as indistinct parazones or diazones, revealing that prisms are obliquely cut in parazones and transversely cut in diazones. Layers of interprisms alternated with rows of prims. c: Prisms extend in a undulating pattern and are transversally cut. d: Incisal region: Most of prisms are transversally cut. But a few of prisms are almost longitudinally cut. e: Parazones and diazones alternated with each other. Layers of interprisms alternated with rows of prisms. f: Parallel prisms are transversally cut. Thin interprisms surround the prisms. (bar = 10 μm, 1000×)

In the middle regions, the prisms decussated in groups (called HSBs) with abundant interprisms alternating layer by layer (Figs. 3 and 4b, and 4e). One of decussating prism groups was sectioned along their long axis in the longitudinally sectioned surfaces with or without a 45-degree bevel (Fig. 3). The decussating prism groups were almost transversely sectioned in horizontally sectioned surfaces without a 45-degree bevel (Fig. 4b). However, decussating prism groups were obliquely cut on both horizontally sectioned surfaces with a 45-degree bevel and tangential sectioned surfaces (Fig. 4e).

**Fig. 5:**
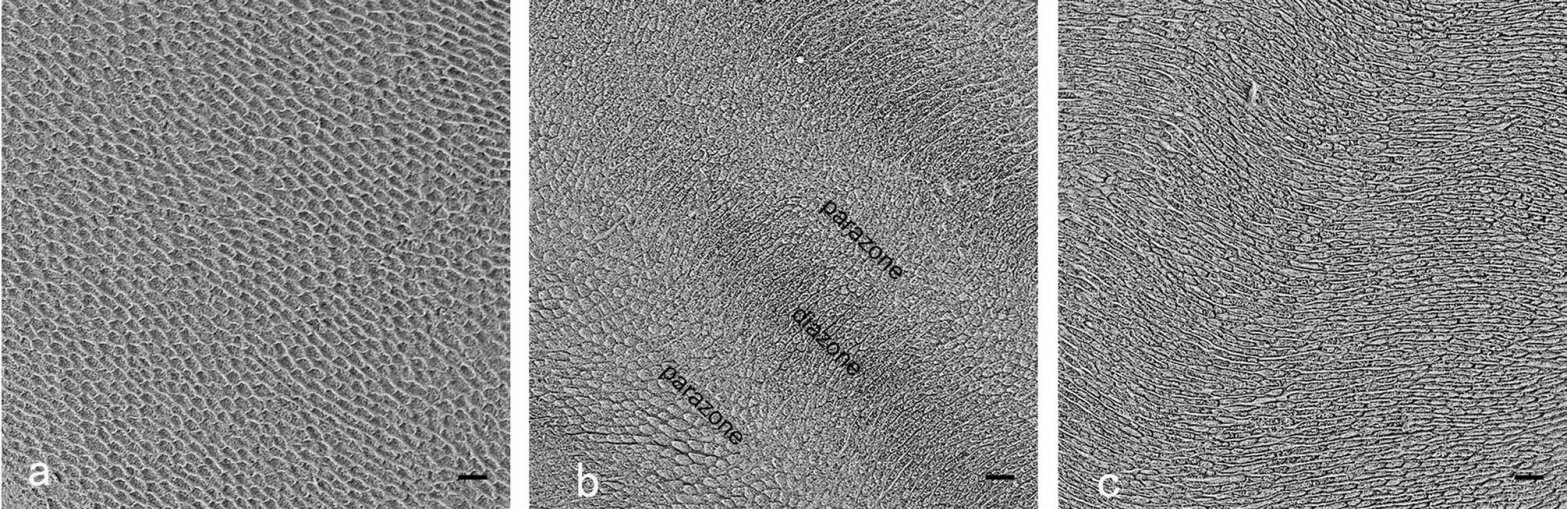
SEM micrographs of tangential enamel sections in three regions. a: Incisal third: the prisms are cut transversally. b: Middle third: The paraozones and diazones of HSBs is not obvious. Prisms are cut transversally or obliquely. Thin interprisms alternate with prisms. c: Cervical third: the prisms are transversally cut and surrounded with interprisms. (bar = 10 μm, 1000)

The prisms extended from the enamel-dentin junction (EDJ) to the labial surface in a parallel pattern in the incisal and cervical regions. The much thinner interprisms were observed in those regions. Furthermore, the parallel prisms in the incisal regions showed a little larger diameter than those in the cervical regions (Figs. 4 and 5).

### Fractured surfaces of resin-bonded-enamel samples

**Fig. 6:**
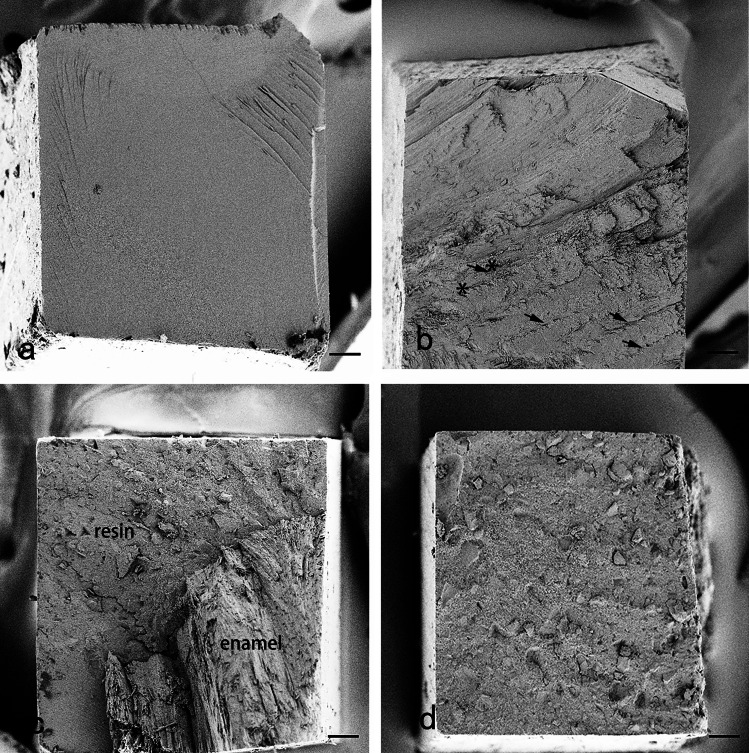
SEM fractographs of the resin-bonded enamel samples. a: The adhesive failure reveals a thin layer of adhesive remaining on the fractured surfaces of the enamel side of the tangential (labial) resin-bonded enamel sample. Several prisms exfoliated from the bonded surface (*). b: The enamel cohesive failure reveals enamel remnants on the resin side of the longitudinally-sectioned resin-bonded enamel sample. Rows of prisms exfoliated from the enamel which are mostly parallel to each other (arrows). c: The mixed failure horizontally-sectioned resin-bonded enamel sample reveals both resin and enamel prisms remained on fracture surface. d: The resin cohesive failure reveals resin remnants on the enamel side of the tangentially-sectioned, resin-bonded enamel sample. (bar = 100 μm, 150×)

SEM micrographs revealed the microstructure of the fractured surfaces of the four failure modes. SEM fractography of the adhesive failure showed a thin adhesive layer remaining on most fractured surfaces at the enamel side (Fig. 6a). SEM fractography of the cohesive failure in the longitudinally sectioned resin-bonded enamel sample revealed that rows of prisms exfoliated from the fracture surface of the enamel side (Fig. 6b). The mixed failure mode revealed both resin and enamel prisms remained on fracture surface (Fig. 6c), and resin cohesive failure reveals resin remnants on both enamel and resin sides (Fig. 6d).

## Discussion

The aim of the study was to investigate the effect of enamel spatial microstructure on the enamel bonding. The enamel bonding is correlated with the microstructures of prism-interprisms, and parallel bonding to prisms may lead to weak enamel bond strength. Thus, when preparing the cavity for composite restoration, the margin configuration should be designed with enamel prisms vertical to the bonded surfaces.

**Fig. 7:**
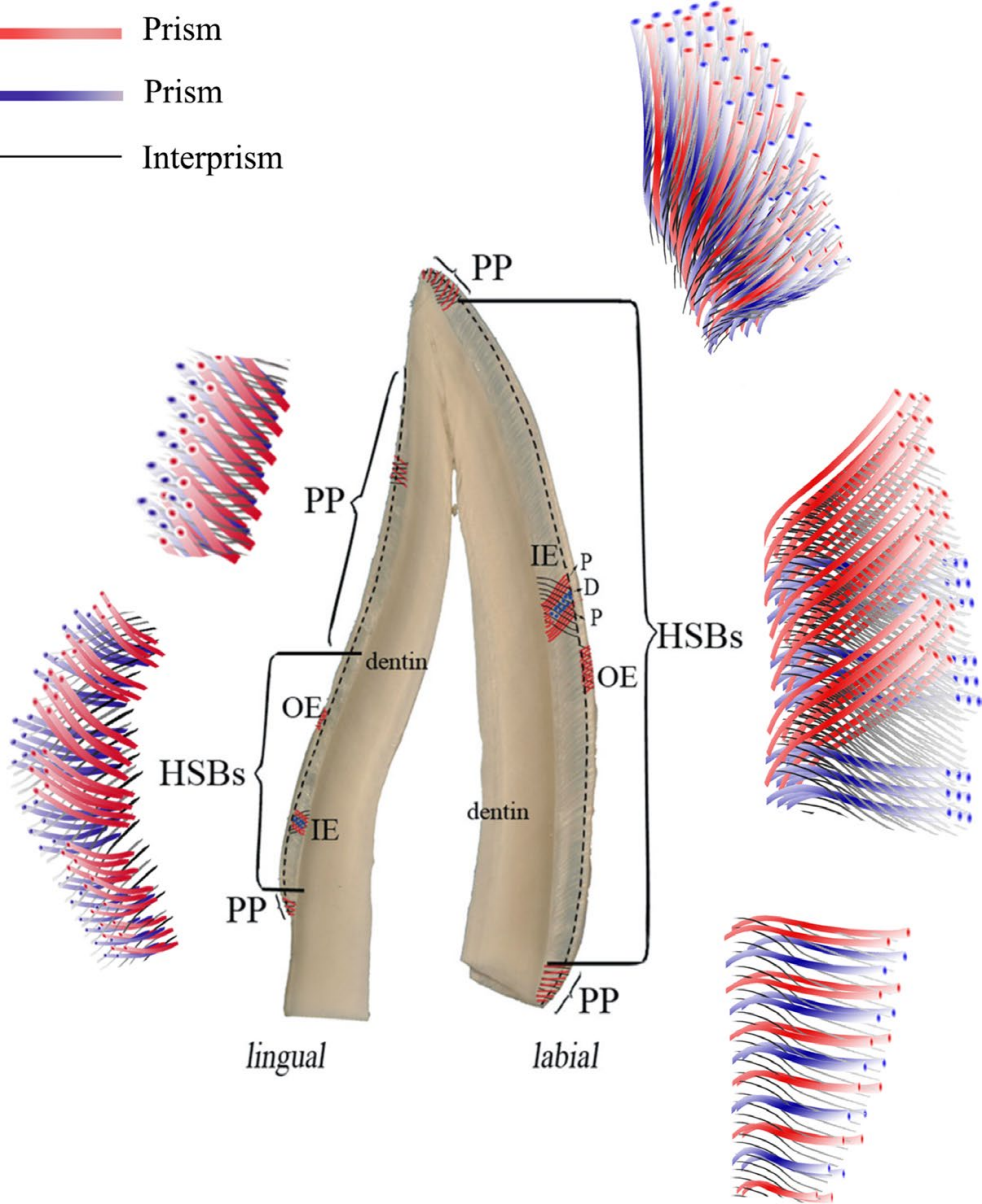
Schematic diagram of prism-interprisms microstructure in different regions

On the basis of SEM observation, we found the microstructures of prism-interprisms varied from regions (Fig. 7**)**, and the enamel bonding was found different, too. The longitudinally sectioned resin-bonded samples had the lowest enamel MTBS. The longitudinally sectioned surfaces showed prisms of parazones of HSBs, parallel to the surfaces (Fig. 3). In contrast, horizontally or tangentially sectioned resin-bonded samples showed much higher values of enamel MTBS. The horizontally or tangentially sectioned surfaces showed prisms of both prazones and diazones of HSBs, perpendicular or oblique to the surfaces (Figs. 4b and 5b). Transversally cut prisms are more easily etched for the permeation of composite resin [32]. Thus, we inferred that parallel bonding to prisms led to weak enamel bond strength, and totally or nearly vertical bonding to prisms produced strong-enamel bond strength. Additionally, the cohesive failure in enamel occurred mostly in the longitudinally sectioned resin-bonded samples with or without a bevel. Abundant interprisms alternated with layered prisms. Cohesive forces between prisms and interprisms are much weaker than those of prisms [16–18, 33, 34]. Rows of prisms parallel to the longitudinally sectioned surfaces were found exfoliating from the fractured surfaces (Fig. 6b). The weak cohesive strength between prisms and interprisms may be another reason for the low enamel MTBS of longitudinally sectioned resin-bonded samples. This finding further confirmed that the enamel bond strength was related to the orientation of the prisms. Therefore, when preparing the cavity for composite restoration, the margin configuration should be designed to favor enamel bonding by cutting enamel prisms almost vertical to their long axis rather than parallel to their long axis.

**Fig. 8:**
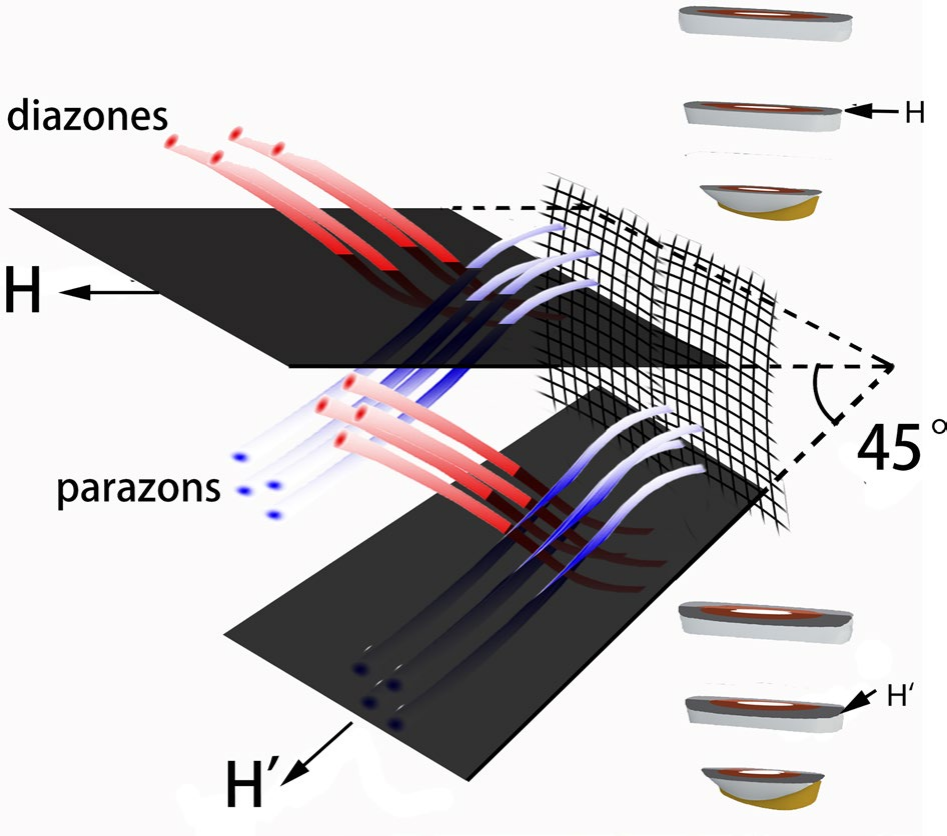
Three-dimensional diagram illustrating the relationship of horizontally cutting direction (H) and horizontally cutting direction with a 45-degree bevel (H’). Both groups of prisms are almost transversally cut when sectioned in a horizontally cutting direction. Prisms of one group are longitudinally cut when sectioned in a horizontally cutting direction with a 45-degree bevel. (D: dentin, E: enamel, Lattice: Enamel-dentin junction. Red and blue cylindrical shapes indicate prisms of parazone and diazone, respectively)

As shown, the preparation of a 45-degree bevel could not improve the enamel-bond strength [26]. The bevel plus the longitudinally sectioned surfaces did not improve the enamel MTBS. The result was supported by similar SEM micrographics of longitudinally sectioned surfaces with or without the 45-degree bevel. However, the 45-degree bevel preparation could slightly decrease the enamel MTBS of horizontally sectioned resin-bonded samples. It might be linked to the enamel microstructure that the parazones and diazones of HSBs decussated with each other at nearly 90 degrees (Figs. 3 and 4, and 5) when diazones (parazones) extended incisally, and the others extend cervically (Fig. 3). Although the tooth crown was horizontally cut, the prisms of both parazones and diazones should be cut vertically to their long axis (Fig. 8). A 45-degree bevel was then prepared cervically on half of the horizontally sectioned surfaces (Fig. 1). Due to the bevel preparation, the prisms of groups extending cervically were cut along their long axis (Fig. 8). As discussed, parallel bonding to prisms leads to weak enamel-bonding strength [13, 14, 17, 18]. A lower enamel MTBS was found at horizontally sectioned resin-bonded samples with a 45-degree bevel. Hence, we do not recommend a 45-degree bevel for the cavity margin preparation. Considering the esthetic benefit of the bevel [1], it is necessary to find a more suitable beveling angle.

The results revealed that the enamel bond strength varied with different regions. The incisal region had the lower enamel MTBS than the middle and cervical regions. This is inconsistent with other studies reporting that the cervical enamel has lower bond strength than the mid-coronal enamel because of its aprismatic structure [15]. However, the aprismatic structure was removed in this study, and many transversally cut prisms are exposed at the middle and cervical regions (Figs. 4 and 5). Moreover, the results revealed that the incisal region had much larger prisms than the middle and cervical regions (Figs. 4 and 5). Thus, the number of transversally cut prisms per surface in the incisal region is a little lower than that in the middle and cervical regions, and it could explain the lower enamel-bond strength was found in the incisal region.

In this study, the bonding ability of all four self-etch adhesive systems was influenced by the region and section direction, without significant differences found among them (*P* > 0.05), consistent with previous reports [35, 36].

Regarding the limitations of the present study, more newer version self-etching adhesive or etch and rinsed adhesive were not investigated. Additionally, other variables have been demonstrated in to have an influence on bond strength of composite materials, such as the application of bleaching agents [37], fluorides [38], cleaning methods [39] and substrate contamination [40]. Therefore, enamel three-dimension spatial microstructure should be evaluated in the future also in combination with these variables.

## Conclusion

The enamel bonding was related to the spatial prism-interprisms microstructures. The regions and sectioned directions need to be taken into consideration when preparing tooth for filling or restoration. The parallel bonding to prisms resulting in weak enamel bonding should been avoided as far as possible. A 45-degree bevel for the cavity margin preparation had no improvement on the enamel bonding.

## Data Availability

All data generated or analyzed during this study are included in this published article.
